# The Impact of Oral‐Gut Microbiota Structure and Its Manipulation on RA Development and Management

**DOI:** 10.1002/fsn3.72090

**Published:** 2026-07-31

**Authors:** Parisa Ahmadi, Mahmoud Mahmoudi, Sina Mozaffari‐Jovin, Ramiar Kamal Kheder, Tola Abdulsattar Faraj, Samaneh Mollazadeh, Jafar Hajavi, Hanieh Kolahdooz, Seyed‐Alireza Esmaeili

**Affiliations:** ^1^ Immunology Research Center Mashhad University of Medical Sciences Mashhad Iran; ^2^ Immunology Department, Faculty of Medicine Mashhad University of Medical Sciences Mashhad Iran; ^3^ Department of Medical Genetics, School of Medicine Mashhad University of Medical Sciences Mashhad Iran; ^4^ Medical Genetics Research Center Mashhad University of Medical Sciences Mashhad Iran; ^5^ Medical Laboratory Science Department, College of Science University of Raparin Rania Sulaymaniyah Iraq; ^6^ Department of Medical Analysis, Faculty of Applied Science Tishk International University Erbil Iraq; ^7^ Natural Products and Medicinal Plants Research Center North Khorasan University of Medical Sciences Bojnurd Iran; ^8^ Department of Basic Sciences, Faculty of Allied Medicine Gonabad University of Medical Sciences Gonabad Iran

**Keywords:** fecal microbiota transplantation, gut microbiota, immune response, oral microbiota, probiotic, RA

## Abstract

Rheumatoid arthritis (RA) is a prevalent autoimmune disease shaped by genetic susceptibility and environmental triggers, particularly the microbiota. Numerous studies have documented substantial structural differences in the oral‐gut microbiota between patients with RA and healthy individuals. Emerging evidence suggests that upregulation of specific pathobionts—including *Porphyromonas gingivalis* , *Aggregatibacter actinomycetemcomitans* , and *Prevotella copri* —coupled with autoantigen mimicry and inflammatory cascades, may contribute to RA initiation at extra‐articular sites. Conversely, several immunoregulatory bacterial species, such as *Bacteroides fragilis* , *Prevotella histicola* , and *Clostridium* spp., are depleted in patients with RA. Importantly, nutritional factors (including, minerals, vitamins, fiber, flavonoids and polyphenols) and dietary patterns (e.g., Western diet, high‐fat diet, Mediterranean diet, fasting, protein‐ or carbohydrate‐rich diets) profoundly shape oral‐gut microbial composition, and dietary interventions can either potentiate or suppress these microbial populations. Accordingly, recent management strategies have focused on manipulating dysbiosis through probiotic supplementation (e.g., immunomodulatory *Lactobacillus* strains), fecal microbiota transplantation (FMT), antibiotic administration, and targeted nutritional interventions that support probiotic efficacy and restore eubiosis. This review discusses the characteristic microbiota structure in patients with RA and evaluates emerging therapeutic strategies—FMT, antibiotics, probiotics, and nutrition—as integrated approaches for RA management.

## Graphical abstract

(A) Schematic representation of oral–gut microbiota composition in health and rheumatoid arthritis (RA), highlighting the enrichment of beneficial microorganisms (green) under healthy conditions and the expansion of pathogenic microorganisms (orange) in RA‐associated dysbiosis. (B) Microbiota manipulation strategies, including dietary and nutritional interventions, fecal microbiota transplantation (FMT), and probiotics/antibiotics, aimed at restoring microbial homeostasis. (C) Restoration of oral–gut microbial balance contributes to RA prevention and alleviation by reducing inflammation and improving joint health.

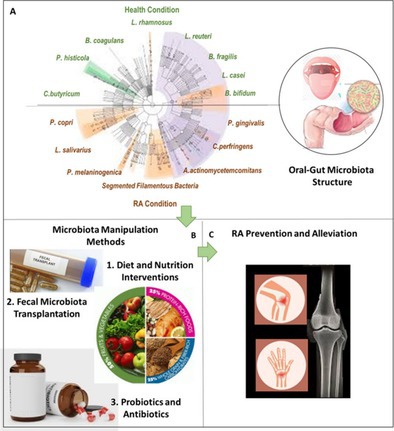

## 1 Introduction

RA is a chronic systemic inflammatory disease affecting 0.1%–2.0% of the global population and is influenced by both genetic and environmental factors (Almutairi et al. 2021; Ebringer et al. 2010; Zafari et al. 2021, 2019). Among environmental factors, the microbiota—the community of microorganisms inhabiting the human body—plays a major role in immune regulation through metabolites such as short‐chain fatty acids (SCFAs), enzymes, vitamins, and lipopolysaccharides (LPS) (Adak and Khan 2019; Escher and Taminiau 2001; Jandhyala et al. 2015). The microbiota is shaped by factors including diet, hygiene, smoking, and medication use, while dysbiosis is increasingly recognized as a contributor to immune dysregulation and autoimmune diseases such as RA, inflammatory bowel disease, Sjögren's syndrome, and systemic lupus erythematosus (Ahmadi, Janzadeh, et al. 2025; Berg et al. 2020; Deng et al. 2022; Ferrillo et al. 2023; Nikitakis et al. 2017). Importantly, microbiota‐driven immune activation may precede the clinical onset of RA, suggesting dysbiosis as a potential initiator of disease. The close coexistence of microbiota and immune cells within the oral and gastrointestinal systems forms the oral‐gut microbiota–immune axis, which plays a central role in systemic inflammation and immune homeostasis (Picchianti Diamanti et al. 2020).

Both pathogenic and protective bacteria within the oral‐gut axis contribute to RA pathogenesis. The gastrointestinal tract harbors one of the densest microbial ecosystems in the body, dominated mainly by Firmicutes and Bacteroidetes, while the oral cavity contains nearly 700 bacterial species (Adak and Khan 2019; Hold et al. 2002; Jandhyala et al. 2015; Yamashita and Takeshita 2017). Evidence linking microbiota to RA includes detection of bacterial LPS in swollen joints, circulating antibodies against oral and gut bacteria, and altered oral‐gut microbial composition in patients with RA (Brusca et al. 2014; Taneja 2014). Pathogenic bacteria such as *Prevotella copri* , *Porphyromonas gingivalis* , *Aggregatibacter actinomycetemcomitans* , and segmented filamentous bacteria (SFB) promote inflammation through mechanisms including Th17 induction, molecular mimicry, protein citrullination, anti‐CCP antibody production, CTLA‐4 downregulation, and activation of autoreactive T cells (Looh et al. 2022; Ma, Zhang, et al. 2021; Nii et al. 2023; Woo et al. 2021). In contrast, beneficial bacteria such as *Bacteroides fragilis* , *Prevotella histicola* , and *Clostridium* spp. help maintain immune tolerance by promoting regulatory T (Treg) cells and suppressing pro‐inflammatory Th17 responses through SCFA production and polysaccharide A signaling (Balakrishnan et al. 2021; Round and Mazmanian 2010).

**FIGURE 1:**
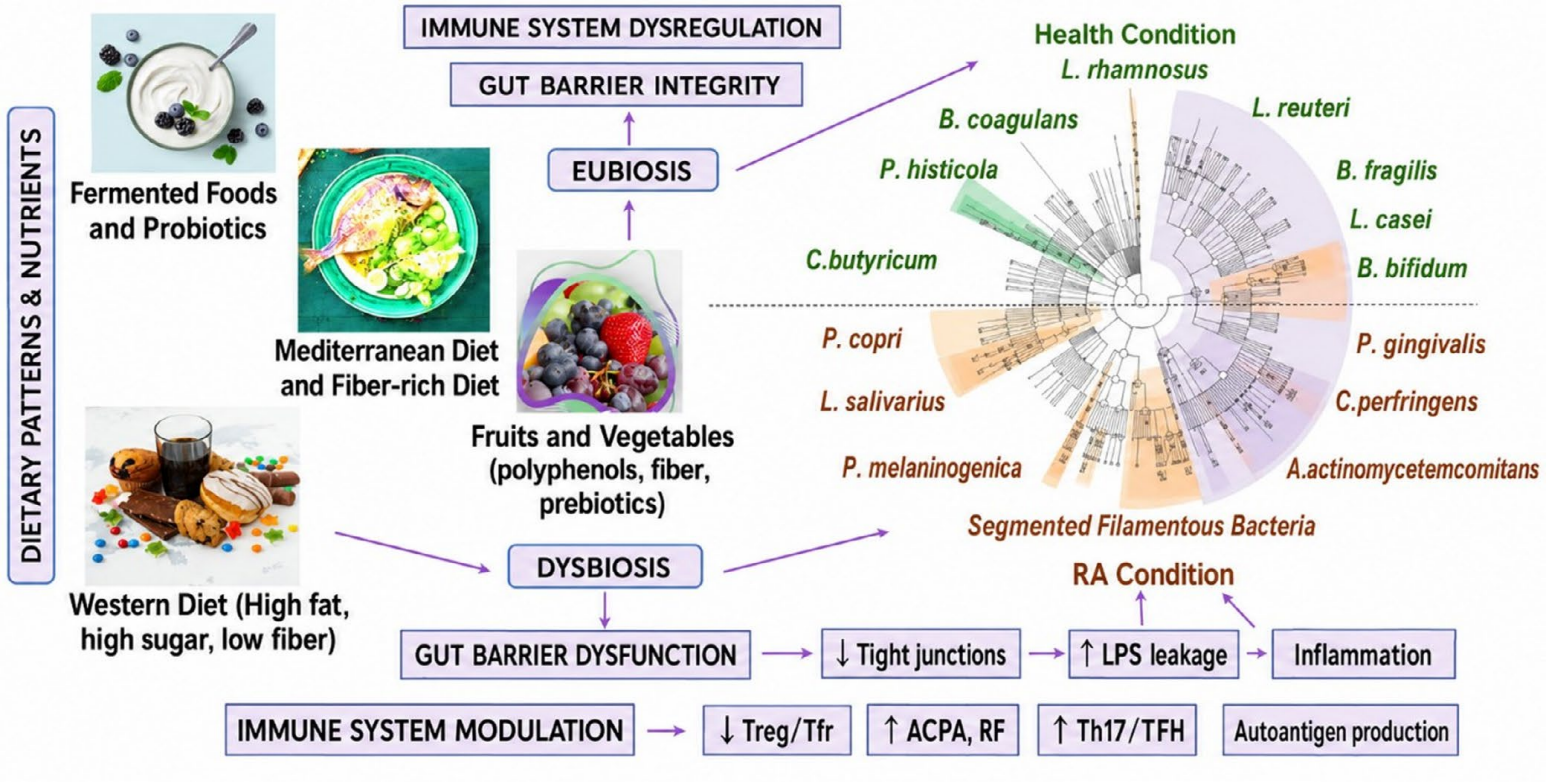
Schematic illustration of how distinct dietary patterns shape gut microbiota composition, intestinal barrier integrity, and immune regulation in rheumatoid arthritis (RA). Western diet (high fat, high sugar, low fiber) promotes dysbiosis, characterized by overgrowth of pathobionts such as *Porphyromonas gingivalis* , *Prevotella copri* , *Aggregatibacter actinomycetemcomitans* , and *Clostridium perfringens. Dy*sbiosis disrupts gut barrier function (↓ tight junctions, ↑ LPS leakage), leading to immune dysregulation with ↑ Th17/Tfh responses, ↓ Treg/Tfr, and increased production of ACPA, RF, and autoantigens. In contrast, fermented foods, vegan diet, and Mediterranean diet (rich in fiber, polyphenols, omega‐3 fatty acids, and fermented foods) supports eubiosis, characterized by abundance of beneficial commensals such as *Bacteroides fragilis* , *Lactobacillus casei* , *Bifidobacterium bifidum* , *Prevotella histicola* , and butyrate‐producing *Clostridium* spp. Eubiosis preserves gut barrier integrity, promotes immune tolerance (balanced Th17/Treg, Tfh/Tfr), and suppresses autoantibody and autoantigen formation, thereby protecting against RA development and progression.

Nutrition is a major determinant of microbiota composition and can promote either eubiosis or dysbiosis (Berg et al. 2020; Buccigrossi et al. 2013; Mei et al. 2021; Sedghi et al. 2021; Taneja 2014). Plant‐based fibers support SCFA‐producing bacteria and improve gut barrier integrity, whereas Western diets rich in fat and sugar favor pro‐inflammatory taxa, increase intestinal permeability, and enhance LPS translocation, which correlates with RA disease activity (Benus et al. 2010; Brusca et al. 2014; Buccigrossi et al. 2013; Taneja 2014). Similarly, dietary habits influence oral microbiota composition, with fermentable carbohydrates promoting dysbiosis while nitrate‐rich vegetables support beneficial nitrate‐reducing bacteria (du Toit et al. 2024; Jurakova et al. 2023). Antibiotics may also alter microbiota composition and contribute to immune sensitization and dysbiosis (Armstrong et al. 2020) (Figure 1).

Microbiota‐targeted therapies for RA include probiotics, fecal microbiota transplantation, and antibiotics (Kloppenburg et al. 1994). Probiotic strains such as *Lactobacillus* and *Bifidobacterium* have shown beneficial effects in reversing dysbiosis and improving RA outcomes (Ashraf and Shah 2014; Fan et al. 2020; Kouchaki et al. 2017). The effectiveness of these approaches is strongly influenced by nutritional support, as prebiotic‐rich diets help sustain beneficial bacteria and stabilize microbial balance following probiotic administration, FMT, or antibiotic therapy (Armstrong et al. 2020). In addition, dietary polyphenols and Mediterranean‐style diets have demonstrated anti‐inflammatory and microbiota‐modulating effects (Ahmadi 2026; Ahmadi, Taherkhani, et al. 2025; Taherkhani et al. 2024). Compounds such as green tea catechins, curcumin, garlic, cranberry, ginger, and arginine‐rich foods can suppress pathogenic bacteria while promoting beneficial commensals, thereby enhancing microbiota‐targeted therapies and supporting immune homeostasis (Bai et al. 2021; Betts and Wareham 2014; Häger et al. 2019; Hashim et al. 2025; Lou et al. 2024; Picchianti Diamanti et al. 2020) (Figure 1).

This article reviews differences in oral‐gut microbiota between patients with RA and healthy individuals, immune regulation by key pathogenic and protective bacteria, and microbiota‐based RA therapies—especially nutritional strategies. A literature search in PubMed and Google Scholar used terms such as “RA,” “oral/gut microbiota,” “dysbiosis,” “probiotic,” “fecal microbiota transplantation,” “antibiotic,” “diet,” and “nutrition.” Studies included in vitro, animal, and clinical investigations. The review highlights how oral‐gut dysbiosis contributes to RA initiation and progression, how diet modulates microbial composition and immune balance, and the implications for microbiota‐targeted therapies. Understanding these interconnected mechanisms is essential for developing integrated, nutrition‐aware therapeutic strategies for RA prevention and management.

## 2 Direct Effects of Nutrition on RA

Several systematic reviews and meta‐analyses have evaluated dietary interventions in RA, although overall evidence quality remains low to moderate. Healthy dietary patterns, including anti‐inflammatory, Mediterranean, and Healthy Eating Index diets, were associated with a 16%–46% lower risk of RA (Joerns et al. 2025). While one meta‐analysis found no significant association between Mediterranean diet adherence and RA incidence (Gianfredi et al. 2026), another large cohort study and meta‐analysis reported a protective effect (Hu et al. 2025). Vegetarian and vegan diets also showed modest but significant pain reduction, although without significant improvement in disease activity or physical function (Jensen et al. 2025).

Specific nutrients may also influence RA risk and outcomes. Dose–response analyses showed that each 80 g/day increase in fruit intake reduced RA risk by 5%, while each 30 g/day increase in cereal intake reduced risk by 3% (Dong et al. 2024). Vitamin D supplementation (600–800 IU/day) reduced autoimmune disease risk by 45%, although pooled analyses across all doses were not significant (Low et al. 2024). Additional supplements reported to improve DAS28 scores included zinc sulfate, copper sulfate, selenium, lipoic acid, turmeric, pomegranate extract, chamomile, cranberry extract, vitamins A, B6, C, D, E, and K, as well as fatty acids (Turk et al. 2023).

Conversely, some dietary factors may increase RA risk. Tea intake increased risk by 4% per additional daily cup, and coffee consumption showed non‐linear associations with RA (Dong et al. 2024). Dietary interventions also improved cardiovascular markers by reducing total cholesterol (−0.36 mmol/L) and LDL cholesterol (−0.20 mmol/L), although no significant effects on muscle or fat mass were observed after 2–4 months (Olsen et al. 2024).

Overall, current evidence suggests that healthy dietary patterns, fruits, cereals, vitamin D, and selected supplements may reduce RA risk or improve disease activity, whereas tea and coffee may increase risk. However, more high‐quality randomized controlled trials are needed to confirm these findings and clarify implications for microbiota‐targeted and nutrition‐based RA therapies.

## 3 Indirect Effects of Nutrition on RA via Oral and Gut Microbiota Modulation

Beyond direct effects, diet influences RA by reshaping oral and gut microbiota, thereby modulating systemic immunity. patients with RA exhibit oral dysbiosis characterized by enrichment of anaerobic and pro‐inflammatory taxa including *Lactobacillus salivarius* , *Atopobium*, *Leptotrichia*, *Prevotella*, *Cryptobacterium curtum* , *Firmicutes*, *Selenomonas*, *Fretibacterium fastidiosum*, *Parvimonas micra* , and *Anaeroglobus geminatus* , alongside reduced *Corynebacterium*, *Streptococcus*, *Neisseria*, and *Haemophilus* (Graves et al. 2019; Scher et al. 2012; Zhang et al. 2015) (Table 1). Some taxa, including *Prevotella melaninogenica* , *Streptococcus noxia*, and *Streptococcus sputigena*, are elevated even in patients with RA without periodontitis, indicating that oral dysbiosis may contribute to RA independently of overt periodontal disease (Scher et al. 2012). Increased ACPA levels in patients with RA with periodontitis further suggest that periodontal inflammation amplifies autoantibody responses (Corrêa et al. 2019) (Table 1).

**TABLE 1:** Studies comparing oral microbiota composition between individuals with RA and healthy controls.

Method	Design	Sample source	Sample size	Main findings	Ref
Metagenome shotgun sequencing	Case–control	Oral cavity	Naïve RA (*n* = 105); HC (*n* = 98)	In individuals with RA: ↑ anaerobic bacteria. In HC: ↑ HACEK complex, ↑ *Porphyromonas gingivalis* peptidylarginine deiminase (PPAD), ↑ *Rothia aeria*	Zhang et al. (2015)
16S rRNA V3–V4 sequencing	Case–control	Oral cavity	RA (*n* = 27); high‐risk for RA (Pre‐RA, *n* = 29); HC (*n* = 23)	Firmicutes abundance: HC<Pre‐RA<RA. Proteobacteria abundance: RA<Pre‐RA<HC. ↑ *Rothia* in Pre‐RA. *Actinomyces*, *Prevotella*, and *Selenomonas*: RA>HC. ↓ Actinobacteria and Patescibacteria in HC. ↓ Defluviitaleaceae in RA and Pre‐RA. ↓ *Filifactor* in Pre‐RA. *Neisseria*, *Haemophilus*, *Parvimonas*, and *Eubacterium yurii* group: RA>HC	Tong et al. (2020)
16S rRNA V3–V4 sequencing; ELISA	Case–control	Oral cavity	Periodontal examination: Pre‐RA (*n* = 18), NORA (*n* = 18), CRA (*n* = 49), HC (*n* = 72). Subgingival microbiota analysis: Pre‐RA (*n* = 5), NORA (*n* = 2), CRA (*n* = 3), HC (*n* = 72)	No significant differences were observed in phylum‐level relative abundance among RA groups. Enrichment of Porphyromonadaceae and *Saccharimonas* spp. was identified in Pre‐RA. ↑ *Streptococcus anginosus* in CRA. Periodontitis prevalence and probing pocket depth (PPD) were comparable across RF groups. High RF titers were associated with ↑ Fusobacteria, Saccharibacteria, Spirochaetes, Spirochaetaceae, *Saccharimonas*, and Porphyromonadaceae	Kim et al. (2022)
16S rRNA V3–V4 sequencing	Case–control	Oral cavity	RA (*n* = 30); HC (*n* = 25)	Significant differences in subgingival microbial clustering were observed between RA and HC groups. ACPAs correlated with *Aminipila butyrica* and *Peptococcus simiae*	Chen et al. (2022)

Abbreviations: ACPA, anti‐citrullinated protein antibody; CRA, control rheumatoid arthritis or a comparative RA group; ELISA, enzyme‐linked immunosorbent assay; HACEK, a group of Gram‐negative bacteria including *Haemophilus*, *Aggregatibacter*, *Cardiobacterium*, *Eikenella*, and *Kingella*; HC, healthy control; NORA, likely referring to established RA without specific additional features, as used in the original study; PPAD, *Porphyromonas gingivalis* peptidyl arginine deiminase; Pre‐RA, individuals at high risk for RA prior to diagnosis; RA, rheumatoid arthritis; Ref, reference.

Dietary factors strongly influence this microbial profile. High‐sugar diets promote RA‐associated genera such as *Prevotella*, *Streptococcus*, *Veillonella*, and *Dialister*, while reducing beneficial commensals including *Neisseria* and *Haemophilus* (Tang et al. 2024). In contrast, Mediterranean‐style diets rich in fiber, polyphenols, omega‐3 fatty acids, and fermented foods are associated with lower RA disease activity and may beneficially modulate oral‐gut microbial ecology (Nikiphorou and Philippou 2023; Picchianti Diamanti et al. 2020). Fermented foods may further enhance microbial diversity and SCFA production, although evidence in RA remains emerging. Ongoing trials such as the TASTY study are evaluating the effects of Mediterranean diets enriched with fermented foods on oral and gut microbiota composition in patients with RA (Charneca et al. 2025) (Figure 1).

Mechanistically, oral bacteria contribute to RA through inflammation, immune activation, and citrullination. Bacterial PAMPs activate TLR signaling pathways, inducing cytokines including TNF‐α, IL‐1, IL‐6, and IL‐8 (Yucel‐Lindberg and Båge 2013). Inflammatory conditions increase peptidyl arginine deiminase (PAD) activity, leading to protein citrullination and generation of neoantigens from extracellular matrix proteins such as collagen and fibronectin (Ogrendik 2009b; Sofat et al. 2014). APC‐mediated presentation of these citrullinated peptides promotes T‐cell activation and ACPA production, contributing to loss of immune tolerance and chronic synovial inflammation (Ceccarelli et al. 2019). Importantly, synergistic interactions among oral bacteria may enhance both inflammation and autoantigen generation, amplifying RA pathogenesis (Sato et al. 2017; Zaiss et al. 2021) (Table 1).

Collectively, these findings highlight that dietary modulation of oral and gut microbiota represents a promising strategy for RA prevention and management, with implications for microbiota‐targeted nutritional interventions and functional food development.

### 3.1 *Porphyromonas gingivalis*

Among oral pathobionts, *Porphyromonas gingivalis* is a gram‐negative anaerobe strongly implicated in RA pathogenesis through its gingipains (Rgp, Kgp) and bacterial peptidylarginine deiminase (PPAD). Rgp generates C‐terminal arginine residues that are subsequently citrullinated by PPAD, producing autoantigens such as fibrinogen and α‐enolase (Cheng et al. 2017). *P. gingivalis* also contributes to RF (RF) formation through Fc‐region IgG degradation, generating neo‐epitopes recognized by RF (Ogrendik 2009b). In addition, it enhances inflammation via C5 convertase activity, promotes NETosis and apoptosis, and releases intracellular proteins such as vimentin for further citrullination (Ahmadi et al. 2023; Johansson et al. 2016). PPAD‐mediated citrullination of LL‐37 further supports immune evasion (Yeoh et al. 2013). However, associations between *P. gingivalis* and RA activity or ACPA titers remain inconsistent across studies, likely due to differences in methodology, disease stage, demographics, and medication use (Lee et al. 2015; Scher et al. 2012; Seror et al. 2015).

Emerging evidence suggests that nutrition substantially modulates *P. gingivalis* virulence and its contribution to RA. Oleic acid, a key Mediterranean diet fatty acid, reduces *P. gingivalis* ‐induced bone destruction and inflammation by increasing pro‐resolving mediators such as Resolvin D4 and 4‐HDHA, while suppressing lipotoxic metabolites and osteoclastogenesis (Döding, Hüfner, et al. 2023; Döding, Zimmermann, et al. 2023; Döding et al. 2025). In contrast, palmitic acid, abundant in Western diets, exacerbates inflammation and bone loss (Figure 1).

Several bioactive food compounds also attenuate *P. gingivalis* ‐mediated pathogenicity. Cinnamaldehyde activates PPARγ/Akt/eNOS and Nrf2/ARE signaling while reducing IL‐6, IL‐8, and TNFα (Ou et al. 2022; Sampath et al. 2026). Liposomal curcumin suppresses the TLR4/MyD88/NF‐κB/NLRP3 inflammasome pathway and ROS generation (Diomede et al. 2021). Omega‐3 PUFAs reduce alveolar bone loss and decrease MMP‐2, MMP‐9, TNFα, and IL‐2 expression (González‐Alva et al. 2024). Probiotic strains such as *Streptococcus salivarius* K12 and M18 inhibit volatile sulfur compounds and exhibit antimicrobial activity against *P. gingivalis* (Yoo et al. 2020). Moreover, calcitriol enhances gingival autophagy and LL‐37 production, attenuating periodontal inflammation (Wang, Huang, et al. 2022). Importantly, Mediterranean diet interventions have reduced salivary levels of *P. gingivalis* , *Prevotella intermedia* , and *Treponema denticola* in overweight/obese individuals (Laiola et al. 2020) (Figure 1).

Collectively, these findings indicate that dietary fatty acids, probiotics, vitamins, and phytochemicals can modulate *P. gingivalis* survival, virulence, citrullination capacity, and host inflammatory responses. Nutritional targeting of oral dysbiosis may therefore represent a promising adjunctive strategy for RA prevention and management, particularly in individuals with periodontitis or increased RA susceptibility.

### 3.2 *Prevotella melaninogenica*

*Prevotella melaninogenica* is a saccharolytic bacterium capable of degrading galactose residues in the IgG Fc region, a mechanism associated with poorer RA prognosis (Ogrendik 2009b). It also promotes Th17‐mediated inflammation by stimulating dendritic cells and inducing IL‐1β and IL‐6 production while suppressing Th1 polarization (Moutsopoulos et al. 2012).

Dietary factors may modulate the abundance and activity of *P. melaninogenica* , thereby influencing RA outcomes. Dietary nitrate from leafy green vegetables such as beetroot and spinach enhances its metabolic activity in the oral cavity, contributing to salivary nitrite production (Burleigh et al. 2018). However, oral colonization by this bacterium has also been linked to RA pathogenesis, highlighting a context‐dependent role. Population‐based differences further suggest dietary influence on colonization patterns, as Swedish individuals consuming traditional European diets showed higher subgingival levels of *P. melaninogenica* than American individuals (Haffajee et al. 2005). In contrast, studies in South African populations identified *P. melaninogenica* as part of the healthy oral microbiome, with limited association between diet and bacterial abundance, indicating that host genetics and immune status also shape its pathogenic potential (Govender et al. 2026).

Overall, *P. melaninogenica* contributes to RA progression through IgG modification and Th17 activation, while diet and host‐related factors may modulate its oral abundance and inflammatory effects, supporting the potential of nutrition‐based strategies to reduce its pathogenicity.

### 3.3 *Aggregatibacter actinomycetemcomitans*

*Aggregatibacter actinomycetemcomitans* contributes to RA pathogenesis through leukotoxin A, which forms pores in neutrophil membranes and induces calcium influx, activating human PAD enzymes and promoting citrullination of host proteins that become RA autoantigens (Gómez‐Bañuelos et al. 2019; Konig et al. 2016).

Nutritional and metabolic factors substantially influence the colonization and pathogenicity of *A. actinomycetemcomitans* . Diabetes mellitus is associated with markedly higher prevalence of this bacterium and other periodontal pathogens (Al‐Obaida et al. 2020; Choi et al. 2025). Likewise, obesity, elevated BMI, hyperlipidemia, and high‐fat diets enhance *A. actinomycetemcomitans* ‐induced periodontal inflammation and alveolar bone loss by disrupting host immune responses (Chen et al. 2014; Leonov et al. 2024; Li et al. 2015).

In contrast, dietary interventions targeting oral microbiota may reduce its pathogenic burden. Diets low in refined sugars, such as the Stone Age diet, decrease *A. actinomycetemcomitans* colonization (Baumgartner et al. 2009). In vitro studies further showed that vitamin D and probiotic‐derived products from *Limosilactobacillus reuteri*, *Lacticaseibacillus rhamnosus*, and *Lacticaseibacillus casei* attenuate *A. actinomycetemcomitans* ‐induced IL‐6 production and cytotoxicity in oral epithelial cells (Zanetta et al. 2025). Calcium supplementation may also protect against infection‐induced bone loss by modulating inflammatory responses (de Albuquerque Taddei et al. 2014).

Collectively, these findings indicate that hyperglycemia, obesity, high‐fat diets, and refined sugar intake may enhance the pathogenic effects of *A. actinomycetemcomitans* , whereas vitamin D, probiotics, calcium, and low‐sugar dietary patterns may help reduce oral dysbiosis and RA‐related inflammatory responses.

### 3.4 *Lactobacillus salivarius*

*Lactobacillus salivarius* is a gram‐positive, facultative anaerobic, lactic acid–producing bacterium commonly found in the human oral cavity and gastrointestinal tract (Abdelgadir et al. 2024; O'Shea et al. 2012). Although several *Lactobacillus* species are traditionally considered beneficial probiotics, increasing evidence suggests that *L. salivarius* may act as a context‐dependent pathobiont in RA.

Studies of untreated patients with RA have reported increased abundance of *L. salivarius* in oral and gut microbiota compared with healthy controls (de Oliveira et al. 2017). Its overrepresentation correlates positively with disease severity markers, including CRP, ESR, and IgG levels (Diamanti et al. 2016; Elsouri et al. 2021; Sultan et al. 2019). Mechanistically, *L. salivarius* has been shown to stimulate production of pro‐inflammatory cytokines such as IL‐1β, IL‐8, and TNF‐α in peripheral blood mononuclear cells, suggesting activation of innate immune and NF‐κB‐related inflammatory pathways (Ashraf and Shah 2014).

However, the role of *L. salivarius* in RA appears strain‐dependent. In a collagen‐induced arthritis model, certain strains of *L. salivarius* reduced arthritis severity, synovial inflammation, and bone erosion through enhancement of Treg responses, suppression of Th17 cells, and increased IL‐10 production (Liu et al. 2016). These contradictory findings indicate that probiotic effects cannot be generalized at the species level and highlight the importance of strain‐specific characterization in microbiota‐targeted RA therapies.

Therefore, despite belonging to a traditionally probiotic genus, *L. salivarius* should not be uniformly categorized as beneficial in RA and may instead function as a pro‐inflammatory pathobiont under specific host and dietary conditions.

## 4 Gut Microbiota and Immune Dysregulation in RA

RA is characterized by immune imbalance, particularly increased Th17/Treg ratio and elevated IL‐17, IL‐23, IL‐6, and TNF‐α, with reduced TGF‐β (Niu et al. 2012). The gut microbiota plays a central role in regulating these immune responses, and diet is a major factor shaping microbial composition and RA progression (Table 2).

**TABLE 2:** Studies comparing gut microbiota composition between individuals with RA and healthy controls.

Method	Design	Sample source	Sample size	Main findings	Ref
Metagenome‐linked group studies	Case–control	Fecal samples	Total fecal samples (*n* = 212)	In individuals with RA: ↑ *Clostridium asparagiforme* , *Gordonibacter pamelaeae* , *Eggerthella lenta* , Lachnospiraceae bacterium, *Bacteroides* spp., *Prevotella copri* , and acetate‐producing *Clostridium* spp. Gut microbiota showed enrichment of Gram‐positive bacteria and depletion of Proteobacteria and Veillonellaceae. In HC: ↑ *Veillonella*, *Haemophilus* spp., *Klebsiella pneumoniae* , *Bifidobacterium bifidum* , *Sutterella wadsworthensis* , and *Megamonas hypermegale*	Zhang et al. (2015)
Fecal 16S rRNA sequencing	Case–control	Fecal samples	RA (*n* = 66); HC (*n* = 60)	In individuals with RA: ↓ *Lactobacillus*, *Alloprevotella*, *Enterobacter*, *Clostridium sensu stricto‐3*, *Odoribacter*, *Akkermansia*, *Helicobacter*, *Rikenella*, and *Coprococcus 1*. ↑ *Bacteroides*, *Escherichia–Shigella*, *Parasutterella*, *Flavonifractor*, *Sellimonas*, and *Oscillospira*	Sun et al. (2019)
Quantitative fecal 16S rRNA sequencing	Case–control	Fecal samples	Untreated early RA (*n* = 17); HC (*n* = 14)	Both groups exhibited *Ruminococcus*, *Bacteroides*, and *Blautia*. However, *Prevotella*, particularly *P. copri* and *P. stercorea* , predominated in the RA‐associated cluster	Maeda et al. (2016)
Fecal 16S rRNA real‐time qPCR	Case–control	Fecal samples	Early RA (*n* = 15); HC (*n* = 15)	Early RA was associated with ↑ fecal *Lactobacillus* diversity, predominantly *L. salivarius* , *L. iners* , *L. ruminis* , and *L. mucosae*	Liu et al. (2013)
Next‐generation sequencing of bacterial 16S rRNA genes	Case–control	Fecal samples	RA (*n* = 42); HC (*n* = 10)	In individuals with RA: ↑ Lactobacilli phylum and ↓ *Faecalibacterium*, especially *F. prausnitzii* . In HC: *Flavobacterium* genus and *Blautia coccoides* were detected exclusively	Picchianti‐Diamanti et al. (2018)
Fecal 16S rRNA sequencing by PCR	Case–control	Fecal samples	RA (*n* = 40); non‐RA subjects including FDR (*n* = 15) and HC (*n* = 17)	In individuals with RA: ↑ rare taxa including *Actinomyces*, *Eggerthella*, *Streptococcus*, *Turicibacter*, *Collinsella*, and Actinobacteria‐related taxa. ↓ microbial diversity, including *Faecalibacterium*	Chen et al. (2016)
Metagenome‐wide association study	Case–control	Fecal samples	RA (*n* = 82); controls (*n* = 42)	Individuals with RA showed ↑ abundance of *Prevotella denticola* , *Gardnerella vaginalis* , *Prevotella marshii* , *Prevotella disiens* , *Prevotella corporis* , *Prevotella amnii* , and *P. copri*	Kishikawa et al. (2020)
PCR amplification and sequencing	Case–control	Fecal samples	HC (*n* = 30); RA (*n* = 99)	In individuals with RA: ↓ *Bifidobacterium* and *Blautia*; ↑ *Streptococcus* and *Candida*. In HC: ↑ *Aspergillus*, the dominant fungal genus	Lee et al. (2022)
ELISA; real‐time PCR	Cross‐sectional clinical study	Fecal samples	RA (*n* = 87)	Serum LPS‐binding protein (LBP) positively correlated with ESR, CRP, MMP‐3, and IL‐6. Total bacterial counts correlated with endotoxin‐neutralizing capacity (ENC). Inverse correlations were identified between serum LBP and hemoglobin, anti‐Pg‐LPS IgG and RA activity indices, and total bacterial counts with serum LPS and anti‐Pg‐LPS IgA	Kitamura et al. (2022)

Abbreviations: anti‐Pg‐LPS IgA, immunoglobulin A against *Porphyromonas gingivalis* lipopolysaccharide; anti‐Pg‐LPS IgG, immunoglobulin G against *Porphyromonas gingivalis* lipopolysaccharide; CRP, C‐reactive protein; ELISA, enzyme‐linked immunosorbent assay; ENC, endotoxin‐neutralizing capacity; ESR, erythrocyte sedimentation rate; FDR, first‐degree relatives of patients with RA; HC, healthy control; IL‐6, interleukin‐6; LBP, LPS‐binding protein; LPS, lipopolysaccharide; MMP‐3, matrix metalloproteinase‐3; PCR, polymerase chain reaction; qPCR, quantitative polymerase chain reaction; RA, rheumatoid arthritis; Ref, reference.

Pro‐inflammatory dietary patterns such as high‐fat diet and Western diet disrupt gut microbial balance. high‐fat diet reduces butyrate‐producing bacteria, including *Muribaculaceae*, while increasing pro‐inflammatory genera such as *Blautia*, *Oscillibacter*, and *Ruminiclostridium‐9*, leading to intestinal barrier dysfunction, Th17/Treg imbalance, and aggravated arthritis (Yantong Liu et al. 2024; Shi et al. 2024; Zhang et al. 2024). Similarly, Western diet promotes *Haemophilus parainfluenzae* and is associated with increased RA risk (Genc et al. 2025; Polyzou et al. 2025). Although dietary fiber is generally protective, excessive succinate production by *Prevotella copri* under high‐fiber conditions may activate inflammatory macrophages and worsen arthritis (Jiang et al. 2022) (Figure 1).

Conversely, anti‐inflammatory dietary patterns can beneficially modulate microbiota and immune pathways in RA. Mediterranean and plant‐based diets increase beneficial bacteria such as *Faecalibacterium prausnitzii* , reduce *P. copri* , decrease CRP and disease activity, and improve quality of life (Muscogiuri et al. 2026; Nikiphorou and Philippou 2023). Fermentable fibers including pectin and inulin enhance butyrate‐producing bacteria; butyrate restores Th17/Treg balance through AMPK activation and suppresses arthritis (Lou et al. 2024). Magnesium supplementation decreases *Prevotella* while increasing *Bacteroides* and other SCFA‐producing bacteria, promoting Foxp3+ Treg expansion and IL‐10 production (Laragione et al. 2023). Omega‐3 PUFA (2 g/day) lowers TNF‐α, IL‐6, and IL‐1β, while vitamin D modulates gut microbiota and Treg/Th17 balance (Nikiphorou and Philippou 2023; Polyzou et al. 2025) (Figure 1).

Several functional food compounds also show microbiota‐mediated anti‐arthritic effects. Biotin‐free diet reduced *Oscillospira* abundance and attenuated collagen‐induced arthritis (Su et al. 2025). *Angelica sinensis* polysaccharide increased *Lactobacillus*, reduced *Clostridia_UCG‐014*, upregulated the tight junction protein Cldn5, and alleviated joint inflammation (Q. Hu et al. 2022). In addition, *Pueraria lobata* exosome‐like nanovesicles targeted *Ruminococcus gnavus* , reduced phenylethylamine production, and inhibited the PEA‐BTK‐NETs inflammatory axis (Han et al. 2026).

Nutritional timing also influences microbiota and immune regulation. Intermittent fasting increases SCFA‐producing bacteria, promotes autophagy, and reduces joint pain (Barati et al. 2023; Hansen et al. 2026). Time‐restricted eating restores rhythmicity of *Parabacteroides distasonis* and glycitein production, modulating inflammation via the SIRT5–NF‐κB pathway (Ma et al. 2024).

Overall, dietary patterns and food‐derived compounds can reshape gut microbiota, regulate immune signaling, and influence RA severity, highlighting the importance of nutrition‐based and microbiota‐targeted strategies in RA management.

### 4.1 Segmented Filamentous Bacteria

Segmented Filamentous Bacteria (SFB) are gram‐positive, non‐cultivable, spore‐forming commensals with potent immunomodulatory activity in the gut (Caselli et al. 2010). SFB strongly stimulate mucosal immunity by inducing IgA production and activating T cells, B cells, and innate immune pathways (Caselli et al. 2010; Hedblom et al. 2018; Schnupf et al. 2013). Their most prominent immunologic effect is the induction of naïve CD4+ T cells toward the Th17 lineage through IL‐6‐, IL‐23‐, and STAT3‐dependent pathways, leading to increased production of IL‐17 and IL‐22 (Hedblom et al. 2018). Excessive Th17 polarization disrupts the Th17/Treg balance and promotes chronic inflammation associated with RA (Campbell 2014) (Table 2).

Experimental arthritis models have demonstrated that SFB enhance germinal center formation, autoreactive B‐cell activation, autoantibody production, and arthritis severity (Wu et al. 2010). In addition to classical Th17 responses, SFB promote Bcl‐6‐dependent TFH expansion and pathogenic TFH17 differentiation, amplifying germinal center activity and systemic autoimmunity (Block et al. 2016; Fan et al. 2025). SFB also impair T follicular regulatory cell suppressive function through reduced CTLA‐4 expression and altered metabolic signaling, thereby facilitating autoreactive TFH‐ and B‐cell responses (Bates et al. 2021). Moreover, SFB‐induced Th17 cells can migrate through gut–joint and gut–lung axes to peripheral tissues such as the lung, where they contribute to preclinical autoimmunity and chronic inflammatory responses via chemokine pathways including CCL20 (Bradley et al. 2017). Collectively, these mechanisms position SFB as major drivers of Th17/TFH‐mediated autoimmunity and autoantibody generation in RA.

Recent evidence indicates that diet is a critical regulator of SFB abundance and the downstream inflammatory pathways implicated in RA. High‐fat, high‐sugar, and low‐fiber diets alter gut microbial composition, disrupt epithelial barrier homeostasis, and modulate SFB‐dependent Th17 immunity (Kawano et al. 2022; Royer et al. 2023). Fiber‐rich diets, in contrast, promote SFB colonization and support intestinal immune maturation by activating epithelial–immune signaling pathways. Dietary fiber enhances ILC1‐derived IFN‐γ production and ILC3‐mediated IL‐22 signaling, resulting in increased epithelial MHC‐II expression, REG3γ production, antimicrobial peptide secretion, and maintenance of mucosal barrier integrity (Rodriguez‐Marino et al. 2024; Shiratori et al. 2024). Because IL‐17, IL‐22, TNF‐α, and TFH‐mediated germinal center responses are central pathogenic mechanisms in RA, nutritional modulation of SFB may directly influence arthritis severity and systemic autoimmunity.

Conversely, excessive dietary sugar suppresses Th17‐inducing microbiota and promotes expansion of competing pathobionts such as *Faecalibaculum rodentium*, leading to reduced IL‐17‐mediated mucosal protection and impaired immune homeostasis (Kawano et al. 2022). Similarly, purified or chronic low‐fiber diets decrease SFB abundance, reduce IL‐22 and REG3γ production, impair epithelial defense mechanisms, and promote systemic inflammatory activation (Royer et al. 2023; Shiratori et al. 2024). In inflammatory disease models, nutritional interventions such as exclusive enteral nutrition‐like diets antagonized SFB colonization and attenuated TNF‐ and IL‐17A‐mediated intestinal inflammation (Metwaly et al. 2023). These findings collectively suggest that dietary patterns can shape RA pathogenesis by regulating SFB‐driven cytokine networks, including IL‐17, IL‐22, IFN‐γ, TNF‐α, TFH activation, germinal center responses, and autoantibody production.

### 4.2 *Prevotella copri*

*Prevotella copri* is a rod‐shaped, obligate anaerobic Gram‐negative bacterium that is frequently enriched in the gut microbiota of individuals with early‐stage and preclinical RA (Jiang et al. 2022; Nii et al. 2023; Scher et al. 2013). Its expansion is often accompanied by a reduction in beneficial commensals such as *Bacteroides*, reflecting a dysbiotic microbial configuration associated with immune activation and loss of intestinal homeostasis (Scher et al. 2013). Importantly, *P. copri* DNA and 16S rRNA signatures have been detected not only in the gut but also in synovial fluid and peripheral immune compartments of patients with RA, supporting the concept of a gut–joint axis in RA pathogenesis (Nii et al. 2023; Pianta et al. 2017) (Table 2).

From an immunopathological perspective, one of the central mechanisms by which *P. copri* contributes to RA is the induction of pathogenic Th17 immunity. RA‐associated strains activate dendritic cells (DCs) to produce pro‐inflammatory cytokines such as IL‐6 and IL‐23, which are critical for Th17 polarization and stabilization. This leads to enhanced differentiation of Th17 cells and increased secretion of IL‐17A, a key effector cytokine in synovial inflammation, osteoclast activation, and tissue destruction (Nii et al. 2023). In arthritis‐prone SKG mouse models, colonization with *P. copri* exacerbates disease by amplifying intestinal Th17 responses against the arthritis‐associated autoantigen RPL23A, thereby accelerating systemic autoimmune arthritis (Maeda et al. 2016).

In parallel with T‐cell–mediated mechanisms, *P. copri* contributes to humoral autoimmunity. The immunogenic protein Pc‐p27 induces both Th1‐skewed responses and IgA production, which are associated with seropositivity for ACPAs and RF (Pianta et al. 2017; Seifert et al. 2023). Furthermore, molecular mimicry between *P. copri* epitopes and host self‐antigens such as glucose‐6‐phosphate isomerase, GNS, and FLNA may promote breakdown of central and peripheral tolerance, facilitating autoreactive T‐ and B‐cell activation and perpetuation of chronic inflammation (Maeda and Takeda 2019).

Recent evidence also highlights strong strain‐level heterogeneity in the immunogenic potential of *P. copri* . RA‐derived strains elicit significantly higher IL‐6, IL‐23, and IL‐17 responses compared to commensal strains isolated from healthy individuals, indicating that pathogenicity is not uniform across the species but is determined by strain‐specific immunostimulatory properties (Abdelsalam et al. 2023; Nii et al. 2023). This supports a model in which *P. copri* functions as a pathobiont whose inflammatory potential is context‐ and strain‐dependent.

Metabolically, dietary context plays a crucial role in modulating *P. copri* behavior. Under high‐fiber conditions, particularly diets rich in microbially accessible carbohydrates, *P. copri* thrives due to its polysaccharolytic capacity, fermenting complex plant‐derived polysaccharides. In this ecological setting, it produces metabolites such as succinate and fumarate, which can activate macrophage metabolic reprogramming and enhance pro‐inflammatory signaling pathways, contributing to exacerbation of collagen‐induced arthritis in experimental models (Jiang et al. 2022). Conversely, in broader microbial ecosystems enriched with diverse fermentable fibers, *P. copri* –associated metabolic outputs may include SCFAs, which can exert immunoregulatory effects through inhibition of NF‐κB signaling, modulation of histone deacetylases, and regulation of macrophage polarization toward anti‐inflammatory phenotypes.

At the host–microbiome interface, *P. copri* has also been associated with increased epithelial permeability, reduced expression of tight junction proteins (e.g., occludin and claudins), and elevated systemic inflammatory mediators such as IL‐2 and IL‐8, thereby facilitating translocation of microbial products and amplification of systemic immune activation (Gong et al. 2026). These effects further reinforce its role in gut barrier dysfunction and chronic inflammation.

Ecologically, dietary patterns strongly shape the prevalence of *Prevotella* species. Low‐fiber “Western” diets are associated with depletion of *Prevotella* and expansion of *Bacteroides*, whereas high‐fiber diets in non‐industrialized populations promote *Prevotella*‐dominated enterotypes (de Goffau et al. 2022). Importantly, host response to dietary fiber is also microbiome‐dependent; in individuals colonized with *P. copri* , the anti‐inflammatory effects of fiber intake (e.g., reduction in CRP levels) are attenuated, highlighting a microbiome‐mediated modulation of nutritional immunology (Ma, Nguyen, et al. 2021) (Figure 1).

Furthermore, *P. copri* abundance has been shown to correlate with RA disease activity and may decrease following effective immunomodulatory or disease‐modifying antirheumatic therapy (DMARD) treatment, suggesting that it is dynamically linked to systemic inflammatory burden (Andréasson et al. 2024).

Collectively, *Prevotella copri* emerges as a key microbial pathobiont in RA through integrated effects on gut dysbiosis, Th17 axis activation, antigen‐driven and mimicry‐mediated autoimmunity, strain‐specific immunogenicity, epithelial barrier disruption, and immunometabolic reprogramming. Its functional role is highly context‐dependent, shifting along a spectrum from fiber‐adapted commensalism to pro‐inflammatory pathobiont behavior depending on host genetics, microbial ecology, and dietary substrate availability.

### 4.3 *Bacteroides fragilis*

*Bacteroides fragilis* , a gram‐negative gut bacterium, plays an important role in immune maturation and gut‐associated lymphoid tissue development (Mazmanian et al. 2005). Its major immunomodulatory factor, polysaccharide A (PSA), enhances MHC II and IL‐12 expression in dendritic cells, promoting Th1 differentiation and IFN‐γ production. More importantly, PSA activates TLR2 signaling on naïve T cells, inducing FOXP3+ Treg cells, increasing IL‐10 secretion, and suppressing pro‐inflammatory Th1 and Th17 responses (Nagano et al. 2012; Round and Mazmanian 2010; Telesford et al. 2015). In collagen‐induced arthritis models, *B. fragilis* also increased colonic butyrate levels, further supporting Treg differentiation (Furusawa et al. 2013; Zhou et al. 2022). In addition, its LPS reduced TNF‐α and IL‐1β secretion from macrophages and decreased arthritis incidence in collagen antibody‐induced arthritis models (Kitamura et al. 2021). These findings identify *B. fragilis* as a promising microbiota‐based target in RA.

Diet strongly influences the abundance and function of *B. fragilis* , thereby affecting RA‐related immune responses. High‐fat and Western diets increase *B. fragilis* abundance together with gut dysbiosis, inflammation, and metabolic dysfunction (Huang et al. 2024; Shi et al. 2022). Western dietary patterns rich in processed foods and poor in fiber also favor enterotoxigenic *B. fragilis* , which is linked to pro‐inflammatory pathways (Wang et al. 2024). Similarly, pro‐inflammatory diets (E‐DII ≥ 0) increase *B. fragilis* and worsen intestinal health, whereas anti‐inflammatory diets (E‐DII < 0) improve bowel function and reduce inflammatory burden (Costa et al. 2022) (Figure 1).

Conversely, several nutritional interventions beneficially modulate *B. fragilis* . Vitamin D supplementation increases *B. fragilis* abundance and may enhance Treg differentiation and IL‐10 production (Giampazolias et al. 2024). Ramadan intermittent fasting and ketogenic diets also increase *B. fragilis* and improve metabolic and immune parameters, partly through gut–immune axis modulation (Elhag et al. 2025; Wang et al. 2023). Functional food components, including *Perilla frutescens* seed extract, *Canna edulis* fiber, goat milk oligosaccharides, and alpha‐aminobutyric acid, increased *B. fragilis* while reducing inflammation and improving microbial diversity or metabolic signaling pathways such as AMPK/SIRT1 (Chen et al. 2025; Deethai et al. 2025; Felicianna et al. 2025; Santoso and Maliza 2026). In addition, a synbiotic containing *B. fragilis* ATCC25285 and pectin enhanced anti‐inflammatory effects through regulation of gut microbiota, tryptophan metabolism, and intestinal immunity (Wang, Li, et al. 2022).

Dietary interventions in patients with RA also demonstrate microbiota remodeling effects. The Ideal Food Pyramid diet reduced *B. fragilis* and *Prevotella copri* while increasing beneficial taxa such as *Faecalibacterium prausnitzii* and *Akkermansia muciniphila* , correlating with improved clinical outcomes (Kaçar Mutlutürk et al. 2025). Likewise, *Lactobacillus plantarum* supplementation reduced *B. fragilis* and improved gut health (An et al. 2022). Experimental probiotic administration of *B. fragilis* has also shown anti‐inflammatory and metabolic benefits in animal studies (Essa et al. 2025; Hussain et al. 2024; Ku et al. 2026).

Overall, dietary patterns and functional food components can modulate *B. fragilis* and its immunoregulatory mechanisms, including PSA‐mediated TLR2 activation, FOXP3+ Treg induction, IL‐10 production, and Th1/Th17 suppression. These findings support nutrition‐based microbiota modulation as a potential adjunctive strategy for RA management.

### 4.4 Clostridium

*Clostridium* spp. are anaerobic, Gram‐positive, spore‐forming bacteria that critically regulate gut immunity and RA progression (Brasca et al. 2021). Their effects are species‐dependent and strongly influenced by diet and microbial metabolites (Bhutta et al. 2024; Escher and Taminiau 2001). Beneficial butyrate‐producing species, including *Clostridium leptum* , *Clostridium butyricum* , and *Faecalibacterium prausnitzii* (Cluster IV), are depleted in patients with RA (Lee et al. 2019; Rodrigues et al. 2019; Zhan et al. 2026), whereas pathogenic species such as *Clostridium perfringens* , *Clostridium bolteae* , and *Clostridium bartlettii* are enriched and associated with inflammation, Th17 activation, and disease (Jia et al. 2023; Månsson et al. 1971; Plichta et al. 2021) (Table 2).

The major protective mechanism of beneficial *Clostridium* spp. is SCFA production, particularly butyrate (D'Amelio and Sassi 2018; Escher and Taminiau 2001). Butyrate activates FFARs such as GPR109A and inhibits histone deacetylases, promoting FOXP3+ Treg and T follicular regulatory cell differentiation while suppressing Th1/Th17 responses and cytokines including IL‐17, IL‐21, and IFN‐γ (Bhutta et al. 2024; D'Amelio and Sassi 2018; Takahashi et al. 2020). It also strengthens intestinal barrier integrity through upregulation of ZO‐1, occludin, claudin‐1, and mucin production, thereby reducing gut permeability and systemic inflammation (Escher and Taminiau 2001; Takahashi et al. 2020; Yang et al. 2024). Additionally, butyrate‐induced histone acetylation of *CXCR5*, *Bcl‐6*, and *Foxp3* enhances T follicular regulatory‐mediated suppression of autoantibody production in RA (Takahashi et al. 2020) (Table 2).

Dietary patterns strongly shape *Clostridium* composition and function. The Mediterranean diet promotes butyrate‐producing *Clostridia*, including *Clostridium celatum* , and supports a metabolically favorable gut ecosystem (Genc et al. 2025). In contrast, protein‐rich diets increase atypical *Clostridium perfringens* type A, leading to immune complex deposition, synovitis, pannus formation, and cartilage erosion (Månsson et al. 1971). High‐fat diets enrich *Clostridium sensu stricto* cluster 1 and aggravate dyslipidemia and RA‐associated cardiovascular complications (Shi et al. 2019) (Figure 1).

Nutritional and microbiota‐targeted interventions further modulate *Clostridium*‐mediated immune responses. Mixed *Lactobacillus acidophilus* strains reduce pathogenic *Clostridia_UCG‐014* and *Clostridium_sensu_stricto_1* while increasing butyrate and acetate levels, resulting in lower TNF‐α, MMP‐13, anti‐CII antibodies, and arthritis severity (Yang, Hong, et al. 2025). *Bifidobacterium longum* RAPO suppresses Th17 differentiation and IL‐17 production by reducing RA‐associated *Clostridium* species from the Ruminococcaceae family (Jeong et al. 2021). Similarly, fermented foods and plant‐derived polysaccharides exert anti‐inflammatory effects through microbial modulation. *Lactiplantibacillus plantarum*‐fermented hemp seeds restore beneficial taxa such as *Akkermansia muciniphila* and *Lactobacillus johnsonii* , enhance SCFA production, and reduce cartilage damage (Shan et al. 2025). *Lycium barbarum* polysaccharides and licorice (*Glycyrrhiza*) regulate *Clostridium*, *Eubacterium*, and *Faecalibacterium*, improve tight‐junction integrity, and rebalance the Th17/Treg axis (Liu et al. 2023; Yang et al. 2024).

Importantly, microbiota‐derived butyrate itself ameliorates autoimmune arthritis through HDAC inhibition and T follicular regulatory expansion, highlighting the therapeutic potential of diet‐induced microbial metabolites (Takahashi et al. 2020). Collectively, these findings demonstrate that dietary patterns, fermented foods, probiotics, and fiber‐rich botanical compounds can beneficially reshape *Clostridium*‐associated gut ecology and immune signaling in RA, whereas high‐fat and excessive protein diets promote pathogenic *Clostridium* expansion and disease progression. This positions nutritional modulation of *Clostridium* and SCFA pathways as a promising adjunctive strategy for RA prevention and management.

## 5 Probiotics and Diet as Therapeutic Strategies in RA

Probiotics are live microorganisms that confer health benefits through immune modulation, restoration of gut microbial balance, and reinforcement of intestinal barrier integrity (Esmaeili et al. 2017; Rastin et al. 2023; Sarao and Arora 2017). Common probiotic genera include *Lactobacillus*, *Bifidobacterium*, *Streptococcus*, *Leuconostoc*, *Pediococcus*, *Enterococcus*, and *Saccharomyces boulardii*. In RA, probiotics may suppress pathogenic bacteria such as *Porphyromonas gingivalis* , *Aggregatibacter actinomycetemcomitans* , and *Prevotella copri* , while regulating inflammatory pathways and host immunity (Javanmardi et al. 2024; Wieërs et al. 2020).

**FIGURE 2:**
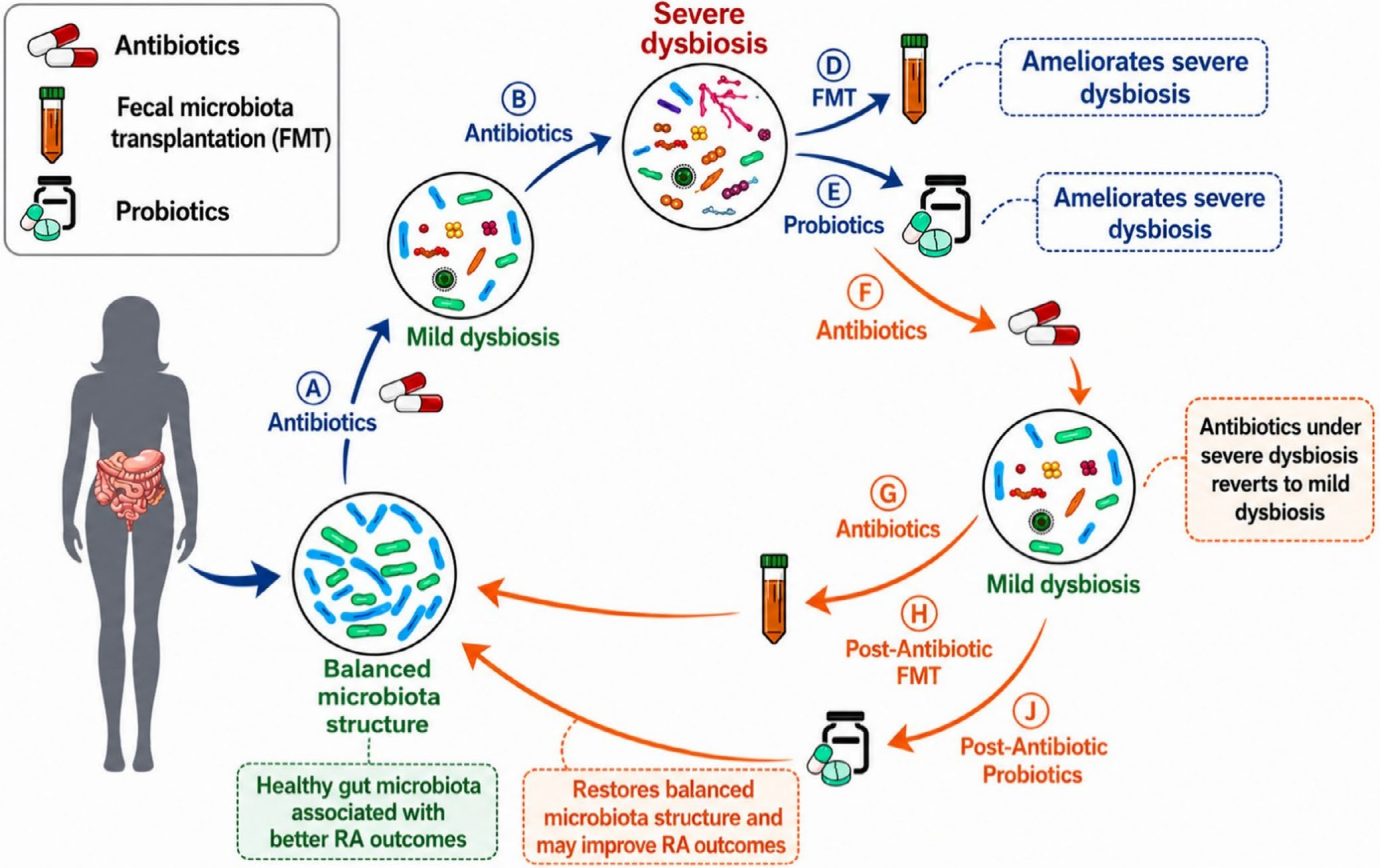
Microbiota manipulation and RA management. Antibiotic administration in a balanced microbiota structure has the potential to induce mild dysbiosis (A, B) that could progress to severe dysbiosis over time (C). RA patients can ameliorate severe dysbiosis via three approaches, including fecal microbiota transplantation (FTM) (D), probiotic administration (E), and antibiotic use (F). Under conditions of imbalanced microbiota, antibiotic administration reverses the severe dysbiosis to mild dysbiosis (F, G). Both fecal microbiota transplantation (H) and probiotic supplementation (I) after an antibiotic intervention could help to restore a balanced microbiota structure.

Most studies in RA have focused on *Lactobacillus* strains. In a clinical trial, *Lactobacillus casei* 01 significantly reduced DAS scores and pro‐inflammatory cytokines (TNF‐α, IL‐6, IL‐12) while increasing IL‐10 after eight weeks (Ahmadi 2025; Vaghef‐Mehrabany et al. 2014). In experimental arthritis models, *L. casei* ATCC334 reduced joint swelling and bone destruction (Pan et al. 2019). Similarly, *L. casei* CCFM1074 and CCFM1075 downregulated IL‐6 and Th17 responses, although only CCFM1074 increased Treg cells and restored gut microbial balance (Fan et al. 2021) (Table 3, and Figure 2).

**TABLE 3:** Effects of probiotic supplementation on clinical outcomes, inflammatory cytokines, and gut microbiota in Rheumatoid Arthritis patients and animal models.

Probiotic supplement	Type	Design	Period	Sample size	Result	JADAD Score (0–5)	Quality Assessment	Ref
*L. casei* 01	Human	Clinical trial	8 weeks	*L. casei* group (*n* = 22) Placebo group (*n* = 24)	↓ TNF‐α, IL‐6, IL‐12 ↑ IL‐10 IL‐1β not significantly affected	3	Moderate (Randomized, double‐blind, but allocation concealment not clearly described)	Vaghef‐Mehrabany et al. (2014)
Probiotic capsules containing *L. acidophilus* , *L. casei* , and *B. bifidum*	Human	Clinical trial	8 weeks	Probiotic group (*n* = 30) Placebo group (*n* = 30)	Improved DAS28 ↓ Serum insulin and hs‐CRP	4	High (Randomized, double‐blind, adequate randomization method, withdrawals described)	Kouchaki et al. (2017)
*L. rhamnosus* GG (LGG)	Human	Clinical trial	12 months	LGG group (*n* = 8) Placebo group (*n* = 13)	Slight ↑ IL‐1β; no significant change in IL‐6, TNF‐α, CRP, ESR; HAQ improvement	3	Moderate (Randomized, double‐blind, but small sample size and high dropout rate?)	Hatakka et al. (2003)
Capsules containing *L. rhamnosus* GR‐1 and *L. reuteri* RC‐14	Human	Clinical trial	90 days	Probiotic group (*n* = 15) Placebo group (*n* = 14)	↓ IL‐8, IL‐12p70, MIP‐1β, GM‐CSF, IL‐1α, IL‐6, IL‐15, TNF‐α	3	Moderate (Randomized, double‐blind, but small sample size)	de los Angeles Pineda et al. (2011)
*Bacillus coagulans* GBI‐30	Human	Clinical trial	60 days	*B. coagulans* group (*n* = 22) Placebo group (*n* = 22)	Improved pain, global assessment, disability, daily activities; ↓ total CRP	3	Moderate (Randomized, double‐blind, but JADAD details not fully reported)	Mandel et al. (2010)
*L. casei* ATCC334	Animal (AIA rats)	Animal trial	30 days	Untreated (*n* = 7), Normal (*n* = 7), MTX (*n* = 7), *L. casei* (*n* = 7)	↓ Joint swelling, bone destruction, arthritis scores, gut dysbiosis	N/A	N/A (Animal study)	Pan et al. (2019)
*Bifidobacterium adolescentis*	Animal (CIA rats)	Animal trial	24 days	Normal (*n* = 8), Untreated (*n* = 8), BA+CIA (*n* = 8), CIA + BA (*n* = 8)	Improved clinical symptoms, rebalanced immune responses, restored dysbiosis	N/A	N/A (Animal study)	Fan et al. (2020)
*Bifidobacterium*, *L. acidophilus* , and *Enterococcus*	Animal (CIA rats)	Animal trial	6 weeks	Control (*n* = 10), model (*n* = 10), probiotic (*n* = 10)	↓ TNF‐α, IL‐6, IL‐1β; restored dysbiosis	N/A	N/A (Animal study)	Jin and Chen (2020)
*L. casei* CCFM1074 and CCFM1075	Animal (CIA rats)	Animal trial	5 weeks	Control (*n* = 8), CIA (*n* = 8), MTX (*n* = 8), CCFM1074 (*n* = 8), CCFM1075 (*n* = 8)	CCFM1074: ↑ Treg cells, rebalanced microbiota, ↓ symptoms; Both: ↓ IL‐6, Th17 cells	N/A	N/A (Animal study)	Fan et al. (2021)
*Bacillus coagulans*	Animal (CFA‐induced arthritis rats)	Animal trial	35 days	Normal (*n* = 8), Inulin (*n* = 8), *B. coagulans* (*n* = 8), Inulin+ *B. coagulans* (*n* = 8), Indomethacin (*n* = 8)	↓ TNF‐α, paw swelling; anti‐inflammatory effect similar to indomethacin	N/A	N/A (Animal study)	Abhari et al. (2016)

Abbreviations: AIA, adjuvant‐induced arthritis; CFA, complete Freund's adjuvant; CIA, collagen‐induced arthritis; CRP, C‐reactive protein; ESR, erythrocyte sedimentation rate; GM‐CSF, granulocyte‐macrophage colony‐stimulating factor; HAQ, Health Assessment Questionnaire; hs‐CRP, high‐sensitivity C‐reactive protein; IL‐10, interleukin‐10; IL‐12, interleukin‐12; IL‐15, interleukin‐15; IL‐1β, interleukin‐1 beta; IL‐6, interleukin‐6; IL‐8, interleukin‐8; MIP‐1β, macrophage inflammatory protein‐1 beta; MPO, myeloperoxidase; MTX, methotrexate; PRE, prebiotic; PRO, probiotic; RA, rheumatoid arthritis; Ref, reference; SYN, synbiotic; Th17 cells, T helper 17 cells; TNF‐α, tumor necrosis factor‐alpha; Treg cells, regulatory T cells.

Other *Lactobacillus* species also demonstrated immunomodulatory effects. Combined administration of *L. rhamnosus* GR‐1 and *L. reuteri* RC‐14 reduced IL‐8, IL‐12p70, GM‐CSF, IL‐6, IL‐15, and TNF‐α in patients with RA (de los Angeles Pineda et al. 2011). A probiotic mixture containing *L. acidophilus* , *L. casei* , and *Bifidobacterium bifidum* improved DAS and reduced hs‐CRP levels (Kouchaki et al. 2017). In contrast, long‐term treatment with *L. rhamnosus* GG showed limited anti‐inflammatory benefit despite modest improvement in HAQ scores (Hatakka et al. 2003). These findings indicate that probiotic effects are highly strain‐specific (Table 3, and Figure 2).

Mechanistically, probiotics influence RA through modulation of the Th17/Treg balance, suppression of NF‐κB signaling, reduction of pro‐inflammatory cytokines, and enhancement of mucosal immune tolerance. Strain‐specific immune responses have also been observed at the molecular level; for example, genomic DNA from *L. acidophilus* and *L. casei* reduced IL‐1β production in PBMCs, whereas *L. plantarum* and *L. delbrueckii* subsp. *bulgaricus* stimulated IL‐1β secretion (Ashraf and Shah 2014) (Table 3).

Beyond *Lactobacillus*, other probiotics have shown therapeutic potential. *Bacillus coagulans* GBI‐30 improved pain, disability, and daily functioning in patients with RA and reduced TNF‐α and paw swelling in arthritic rats (Abhari et al. 2016; Mandel et al. 2010). Likewise, *Bifidobacterium adolescentis* alleviated clinical symptoms and corrected intestinal dysbiosis in CIA rats (Fan et al. 2020) (Table 3).

*Prevotella histicola* is an emerging probiotic with potent anti‐inflammatory properties in rheumatoid arthritis and Inflammatory bowel disease (IBD) (Balakrishnan et al. 2021; Fan et al. 2024). *P. histicola* ameliorates colitis by inhibiting IRE1α‐JNK and NF‐κB pathways, reducing ER stress, and restoring gut barrier integrity (Fan et al. 2024). Additionally, in the gut‐joint axis, *P. histicola* MCI 001 drives a two‐step bacteriological cascade: it degrades complex carbohydrates into acetate and simple sugars, creating a niche for *Allobaculum* expansion. In turn, *Allobaculum* produces high levels of butyrate, an SCFA that suppresses TLR4/NF‐κB/MAPK signaling, promotes Treg differentiation, and enhances tight junction integrity (Balakrishnan et al. 2021; Li et al. 2024; Sun et al. 2025).

From a nutritional standpoint, the growth of *P. histicola* alongside *Allobaculum* is supported by fermentable dietary components. Compounds such as 2′‐fucosyllactose, krill oil, β‐carotene, and high‐esterification pectin promote *Allobaculum* by providing fermentable substrates and increasing SCFA production (Jin et al. 2025; Wang, Wan, et al. 2025; Wang, Xu, et al. 2025; Zou et al. 2025). Probiotic strains ( *L. rhamnosus* , *L. plantarum* + *Astragalus*, *L. casei* ) further enhance this axis via AMPK, Nrf2, and PI3K/Akt pathways (He et al. 2026; Liu et al. 2025; Xia et al. 2025; Yang, Du, et al. 2025). Thus, nutritional interventions supplying fermentable fibers and bioactive compounds can co‐support *P. histicola* and *Allobaculum*, reinforcing gut homeostasis and anti‐inflammatory signaling (Xia et al. 2025; Zou et al. 2025).

Emerging evidence suggests that dietary patterns strongly influence probiotic efficacy and microbiota modulation. Diets rich in fiber, whole grains, vegetables, and fermented foods enhance beneficial microbial metabolites such as SCFAs, strengthen epithelial barrier function, and suppress systemic inflammation. In contrast, high‐fat diets may impair probiotic colonization and exacerbate inflammatory signaling. For example, *L. plantarum* significantly increased only when combined with a balanced diet rich in vegetables and whole grains (Oh et al. 2021). Similarly, probiotic mixtures improved bone and muscle mass under low‐fat diets but not high‐fat diets (Ohlsson et al. 2025). In allergic asthma, a high‐fiber diet synergized with *L. paracasei* to restore immune balance, whereas a high‐fat diet abolished probiotic benefits (Xie et al. 2021).

Although direct evidence in RA remains limited, studies in osteoarthritis and other inflammatory diseases indicate that combining probiotics with targeted dietary interventions may produce synergistic effects through regulation of TLR2/TLR4‐NF‐κB signaling, cytokine production, cartilage metabolism, and gut microbial composition (Korotkyi et al. 2021). Therefore, future RA studies should evaluate integrated nutritional strategies combining anti‐inflammatory diets—such as Mediterranean, low‐glycemic‐index, or fiber‐rich diets—with strain‐specific probiotics. Such approaches may support microbiota‐targeted food product development and personalized nutritional recommendations for RA management (Figure 1).

## 6 Fecal Microbiota Transplantation

Fecal microbiota transplantation (FMT), the transfer of processed stool from a healthy donor to restore gut microbial balance, is emerging as a potential therapy for RA (Wang et al. 2019). Beyond its established efficacy in *Clostridioides difficile* infection, FMT has been investigated in autoimmune and inflammatory disorders due to its ability to reshape gut microbiota and immune responses (Belvoncikova et al. 2022; Vasiliu 2023; Zeng et al. 2021).

Experimental evidence supports a causal role of gut dysbiosis in RA. Pu et al. (2022) demonstrated that fecal transfer from patients with RA to mice induced arthritis and depression‐like behaviors, accompanied by elevated TNF‐α, IL‐6, and Th1/Th2 ratio, alongside reduced Treg cells, highlighting microbiota‐driven immune dysregulation (Pu et al. 2022). In humans, a refractory patients with RA showed marked improvement in DAS28, HAQ‐DI, and RF within 78 days after a single healthy‐donor FMT (Zeng et al. 2021). Although limited to animal studies and one case report, these findings suggest that restoring a diverse microbial ecosystem may provide broader immunomodulatory effects than single‐strain probiotics (Table 4, and Figure 2).

**TABLE 4:** Preclinical and clinical evidence for Fecal Microbiota Transplantation in Rheumatoid Arthritis.

Type	Duration	Design	Sample size	Result	Quality assessment	Ref.
Antibiotic cocktail (ABX)‐treated CIA mice	One fecal transplantation procedure and 39 days follow‐up	Animal trial	FMT from RA patients to ABX‐treated mice (*n* = 10)—FMT from healthy controls to ABX‐treated mice (*n* = 10)—Fecal donation from 7 healthy controls and 6 RA patients	↑ Depression‐like phenotypes, TNF‐α and IL‐6 in RA patients FMT mice ↓ Treg cell percentage in RA patients FMT mice ↑ Th1/Th2 index in RA patients FMT mice	SYRCLE RoB tool: High risk of bias (small sample size, no blinding, no randomization)	Pu et al. (2022)
A 20‐year‐old woman with refractory RA	One fecal transplantation procedure and 78 days follow‐up	Case report	One RA patient Fecal donation from an 8‐year‐old healthy girl	A profound decrease in RF titers, HAQ‐DI, and DAS28 within 78 days after FMT	JBI Checklist for Case Reports: Low quality (single case, lack of control group, limited generalizability)	Zeng et al. (2021)

Abbreviations: ABX, antibiotic cocktail; CIA, collagen‐induced arthritis; DAS28, disease activity score using 28 joints; FMT, fecal microbiota transplantation; HAQ‐DI, Health Assessment Questionnaire Disability Index; IL‐6, interleukin‐6; RA, rheumatoid arthritis; Ref, reference; RF, rheumatoid factor; Th1/Th2 index, T helper 1 / T helper 2 cell ratio; TNF‐α, tumor necrosis factor‐alpha; Treg cells, regulatory T cells.

Current evidence also indicates that diet critically determines FMT success. In ulcerative colitis, combining multi‐donor FMT with an anti‐inflammatory Mediterranean‐style diet rich in fruits, vegetables, legumes, whole grains, fish, and olive oil induced deep remission in 36.4% of patients, whereas FMT alone has shown modest efficacy (Kedia et al. 2022). Similarly, in metabolic syndrome, autologous FMT prevented weight regain only when paired with a high‐polyphenol green‐Mediterranean diet containing walnuts, green tea, and *Wolffia globosa* (Mankai) (Rinott et al. 2021). Subsequent analysis revealed that FMT efficacy depended on diet‐responsive bacteria enriched during the dietary intervention. In contrast, FMT without dietary modification failed completely in Crohn's disease (0% remission), leading investigators to recommend combining FMT with anti‐inflammatory dietary strategies (Kao et al. 2024) (Figure 1).

Given the shared features of RA, ulcerative colitis, and Crohn's disease—including gut dysbiosis, systemic inflammation, and Th17/Treg imbalance—diet‐FMT combination therapies may be more effective than FMT alone in RA. Dietary patterns rich in fiber, polyphenols, fermented foods, and unsaturated fats may enhance beneficial taxa and microbial metabolites such as SCFAs, thereby improving mucosal barrier integrity and reducing inflammatory cytokines. Future RA trials should therefore integrate FMT with targeted nutritional interventions, particularly Mediterranean or high‐fiber diets, to optimize microbiota remodeling and clinical outcomes (Figure 1).

## 7 The Dual Role of Antibiotics in RA

Antibiotics are widely used to treat bacterial infections, but they can also disrupt the gut microbiota and induce dysbiosis (Abramo et al. 2012; Singh et al. 2017). Because gut microbial imbalance is closely linked to immune regulation, increasing attention has been given to the role of antibiotics in RA development and progression (Gobbo et al. 2022; Wieërs et al. 2020). Current evidence suggests a dual and context‐dependent role for antibiotics in RA, acting both as a potential risk factor for disease onset and, in some cases, as a therapeutic strategy after disease establishment (Smith et al. 2022).

### 7.1 Antibiotics as a Risk Factor for RA Onset

Several epidemiological studies have associated antibiotic exposure with an increased risk of RA. A large UK case–control study reported that RA risk increased within 1–5 years after antibiotic use and was strongly associated with both the number of prescriptions and antibiotic class. Individuals receiving more than ten antibiotic courses over five years had nearly double the risk of developing arthritis compared with non‐users (Armstrong et al. 2020). Similarly, another case–control study found that antibiotic use during the five years before RA diagnosis was associated with a 60% higher risk of RA (Sultan et al. 2019) (Table 5).

**TABLE 5:** Summary of epidemiological and interventional studies on antibiotic consumption in rheumatoid arthritis.

Antibiotic type/duration	Design	Sample size	Result	JADAD Score (0–5)	Quality assessment	Ref.
All types	CC	Newly diagnosed RA (*n* = 8482); Healthy controls (*n* = 22,661)	Exposure to antibiotics before RA diagnosis was a significant risk factor	N/A	Good (Large sample size, well‐matched controls)	Armstrong et al. (2020)
All types	CC	RA patients (*n* = 22,677); Healthy controls (*n* = 90,013)	Antibiotic exposure associated with 60% higher odds of RA development; dose/frequency association observed	N/A	Good (Very large sample, dose–response identified)	Sultan et al. (2019)
All types	RCS	133,125 participants	No association between antibiotic usage and risk of RA	N/A	Good (Large cohort, but retrospective design)	Liu et al. (2022)
Tetracycline and clindamycin	DB, PCT	Treatment (*n* = 11); Control (*n* = 10)	45% of RA patients reached ACR20; antibiotic therapy useful for active RA management	3	Moderate (Randomized, double‐blind, but small sample size)	Smith et al. (2011)
Doxycycline	DB	Doxy 100 mg (*n* = 24); Doxy 20 mg (*n* = 18); Placebo (*n* = 24)	Doxycycline‐treated group showed improvement in ACR20 criteria vs. placebo	2	Low‐Moderate (Double‐blind stated, but randomization method not clearly described)	O'Dell et al. (2006)
Doxycycline plus methotrexate	RDB, CT	Doxy + MTX 100 mg (*n* = 24); Doxy 20 mg (*n* = 18); Placebo (*n* = 24)	ACR20 and ACR50 significantly higher in both doxycycline groups vs. placebo	3	Moderate (Randomized double‐blind, controlled trial)	Alarcón (2006)
Minocycline	DB, PCT	Oral mino 200 mg/d (*n* = 109); Placebo (*n* = 110)	Minocycline group showed greater improvement in joint swelling, tenderness, ESR, platelet count, and IgM RF	4	High (Large RCT, double‐blind, placebo‐controlled, clear outcomes)	Trial et al. (1995)
Minocycline	RDB, PCT	Mino 100 mg (*n* = 23); Placebo (*n* = 23)	RA remissions observed in minocycline group; reduced need for DMARD therapy	4	High (Randomized double‐blind, well‐described withdrawal/follow‐up)	O'Dell et al. (1999)
Minocycline	DB, PCT	Mino 100 mg (*n* = 35); Placebo (*n* = 30)	Reduced serum IL‐6 only in minocycline group; decreased IgM‐RF, IgA‐RF, total IgM, total IgA, and IgM‐RF/total IgM ratio	3	Moderate (Double‐blind, but small sample size)	Kloppenburg et al. (1996)
Metronidazole	DB, PCS	Metro 400 mg (*n* = 24); Placebo (*n* = 26)	No improvement in laboratory indices, disease activity, or disease‐modifying properties	3	Moderate (Double‐blind, placebo‐controlled, negative finding)	Marshall et al. (1992)
Roxithromycin	RDB, PCT	Roxi 300 mg (*n* = 50); Placebo (*n* = 50)	Roxithromycin group showed greater ACR20/50/70 achievement; significant only for ACR20	4	High (Randomized double‐blind, placebo‐controlled, adequate sample size)	Ogrendik and Karagoz (2011)

Abbreviations: ACR20, American College of Rheumatology 20% improvement criteria; ACR50, American College of Rheumatology 50% improvement criteria; ACR70, American College of Rheumatology 70% improvement criteria; CC, case–control study; CRP, C‐reactive protein; CT, controlled trial; DB, double‐blind; DMARD, disease‐modifying antirheumatic drug; ESR, erythrocyte sedimentation rate; GI, gastrointestinal; IgA, immunoglobulin G; IgM, immunoglobulin M; IL‐6, interleukin‐6; IV, intravenous; MTX, methotrexate; PCS, placebo‐controlled study; PCT, placebo‐controlled trial; RA, rheumatoid arthritis; RCS, retrospective cohort study; RDB, randomized double‐blind; RF, rheumatoid factor.

The proposed mechanism involves antibiotic‐induced dysbiosis. Different antibiotic classes, including beta‐lactams, glycopeptides, and quinolones, alter gut microbial composition in distinct ways (Patangia et al. 2022). These alterations may promote the expansion of RA‐associated pathobionts such as *Prevotella copri* while reducing beneficial immunoregulatory bacteria, including *Bacteroides fragilis* and *Clostridium* spp. This imbalance may contribute to immune dysregulation and initiation of autoimmunity. Therefore, unnecessary or prolonged antibiotic use, particularly broad‐spectrum antibiotics, should be approached cautiously (Table 5).

### 7.2 Antibiotics as a Potential Therapy for Established RA

Despite their potential role in RA onset, some antibiotics have shown therapeutic benefits in established RA. Before the widespread use of modern disease‐modifying antirheumatic therapies, antibiotics such as minocycline and sulfasalazine were used in RA management (O'Dell 2001; Ogrendik 2009b). Tetracyclines, including minocycline and doxycycline, exhibit not only antimicrobial activity but also anti‐inflammatory, anti‐metalloproteinase, and immunomodulatory effects (Ogrendik 2009a) (Table 5 and Figure 2).

Clinical studies have demonstrated that minocycline can improve disease activity and laboratory markers in patients with RA, including ACR response criteria, ESR, CRP, platelet count, rheumatoid factor, and IL‐6 levels (Kloppenburg et al. 1996; O'Dell et al. 1997; Trial et al. 1995). Doxycycline has also been associated with improvements in ACR20 and ACR50 responses compared with placebo (Alarcón 2006; O'Dell et al. 2006; Pillemer et al. 2003). A meta‐analysis further suggested that minocycline may be more effective than doxycycline in reducing RA disease activity (Table 5).

The beneficial effects of these antibiotics may result from both immunomodulatory actions and partial restoration of dysbiotic microbiota toward a less inflammatory profile. However, findings remain inconsistent. Some placebo‐controlled studies reported no significant benefit of doxycycline or metronidazole on RA symptoms or laboratory (Marshall et al. 1992; Van der Laan et al. 2001). These discrepancies likely reflect differences in study design, patient populations, antibiotic type, dosage, treatment duration, outcome measures, and concomitant therapies (Table 5).

Overall, antibiotics appear to have a context‐dependent role in RA. In healthy individuals, antibiotic‐induced dysbiosis may increase the risk of developing RA, whereas in patients with established disease, selected antibiotics—particularly tetracyclines—may provide therapeutic benefits through anti‐inflammatory and microbiota‐modulating effects.

## 8 Conclusion

Increasing evidence highlights the central role of oral and gut microbiota in the pathogenesis of rheumatoid arthritis (RA) and its associated immune dysregulation. Consequently, microbiota‐targeted strategies—including probiotics, fecal microbiota transplantation (FMT), antibiotics, and dietary interventions—have emerged as promising adjunctive approaches for RA management. Nevertheless, several important knowledge gaps continue to limit their clinical translation and therapeutic standardization.

One major challenge is the absence of a universally accepted definition of “healthy” versus “dysbiotic” microbiota in RA. Microbial composition is strongly influenced by multiple host and environmental factors, including diet, genetics, medication exposure, smoking, geography, and disease stage, leading to considerable variability between studies. Furthermore, most current investigations are cross‐sectional, making it difficult to determine whether dysbiosis represents a cause or consequence of RA. Large longitudinal and multicenter studies are therefore required to identify reliable microbial biomarkers associated with disease onset, progression, and therapeutic response.

Another critical issue involves the strain‐specific effects of microorganisms. Certain bacterial taxa, such as *Lactobacillus salivarius* , *Bacteroides fragilis* , and *Clostridium* spp., may exert either protective or pathogenic effects depending on strain characteristics, host immunity, and environmental context. Future investigations should therefore move beyond species‐level analyses and focus on strain‐level characterization, microbial metabolites, and host–microbe interactions through multi‐omics approaches.

Nutrition also plays a major role in shaping oral and gut microbiota composition and function. Western dietary patterns rich in saturated fats, refined carbohydrates, and processed foods tend to promote dysbiosis, intestinal permeability, and inflammatory signaling. In contrast, Mediterranean‐style, plant‐based, high‐fiber, and fermented‐food diets support beneficial microbial communities, enhance short‐chain fatty acid (SCFA) production, strengthen epithelial barrier integrity, and suppress systemic inflammation. Nutritional compounds including omega‐3 fatty acids, polyphenols, vitamins, prebiotics, and fermented foods may further improve microbial ecology and immune homeostasis, highlighting the therapeutic potential of nutrition‐based microbiota modulation in RA.

Emerging evidence further suggests that combination microbiota‐targeted therapies may provide greater and more durable benefits than isolated interventions alone (Figure 2). Sequential strategies involving targeted antibiotics followed by probiotics or FMT may first reduce pathogenic microbial overgrowth and subsequently restore beneficial commensals and microbial diversity (Figure 2). Concurrent dietary interventions—particularly Mediterranean‐style and high‐fiber diets enriched with prebiotics, polyphenols, and fermented foods—may enhance microbial engraftment, stabilize microbial homeostasis, improve intestinal barrier integrity, and reduce recurrence of dysbiosis. Such integrated approaches may ultimately improve long‐term therapeutic efficacy and immune regulation in RA (Figure 2).

Despite these promising findings, FMT remains insufficiently studied in RA, with unresolved questions regarding donor selection, administration protocols, long‐term safety, and durability of microbial engraftment. Similarly, the dual role of antibiotics requires further clarification, as antibiotics may both induce dysbiosis and provide therapeutic benefit depending on timing, dosage, and disease context.

Overall, RA is a complex systemic autoimmune disease in which genetic susceptibility and environmental factors interact to drive chronic inflammation and immune dysregulation. Comparative studies consistently demonstrate characteristic dysbiosis in RA, including enrichment of pro‐inflammatory and autoimmunity‐associated pathobionts such as *Porphyromonas gingivalis, Aggregatibacter actinomycetemcomitans*, and *Prevotella copri* , alongside depletion of immunoregulatory commensals including *Bacteroides fragilis, Clostridium* spp. Through mechanisms such as molecular mimicry, protein citrullination, intestinal barrier disruption, Th17/Treg imbalance, and persistent cytokine activation, these microbial alterations may contribute to the breakdown of immune tolerance and the development of systemic autoimmunity.

Importantly, the microbiota should not be viewed simply as isolated “good” or “bad” bacteria, but rather as a dynamic ecological network whose collective metabolic and immunologic activities determine disease outcomes. Therefore, the future of RA management likely lies in personalized and multi‐modal microbiota‐based strategies integrating microbiome profiling, precision nutrition, targeted microbial modulation, and immune‐directed therapies. Advancing our understanding of the oral–gut microbiota–immune axis may not only improve RA treatment outcomes but also open new avenues for disease prevention and precision medicine in autoimmune diseases.

## Author Contributions

**Mahmoud Mahmoudi:** supervision. **Hanieh Kolahdooz:** writing – original draft. **Jafar Hajavi:** writing – original draft, investigation, project administration. **Sina Mozaffari‐Jovin:** writing – review and editing. **Samaneh Mollazadeh:** investigation, writing – original draft. **Tola Abdulsattar Faraj:** writing – original draft, writing – review and editing. **Ramiar Kamal Kheder:** writing – review and editing, writing – original draft. **Parisa Ahmadi:** investigation, writing – original draft, project administration. **Seyed‐Alireza Esmaeili:** conceptualization, methodology, supervision, project administration, writing – original draft, writing – review and editing.

## Disclosure

AI use statement: The authors declare that they have not use AI‐generated work in this manuscript.

## Conflicts of Interest

The authors declare no conflicts of interest.

## Data Availability

The data will be made available on request.
